# Circular RNA CircPVT1 Inhibits 5-Fluorouracil Chemosensitivity by Regulating Ferroptosis Through MiR-30a-5p/FZD3 Axis in Esophageal Cancer Cells

**DOI:** 10.3389/fonc.2021.780938

**Published:** 2021-12-13

**Authors:** Wenjian Yao, Jianjun Wang, Fanruo Meng, Zibo Zhu, Xiangbo Jia, Lei Xu, Quan Zhang, Li Wei

**Affiliations:** ^1^ Department of Thoracic Surgery, Henan Provincial People’s Hospital, People’s Hospital of Zhengzhou University, School of Clinical Medicine, Henan University, Zhengzhou, China; ^2^ Department of Thoracic Surgery, Henan University People’s Hospital, Henan Provincial People’s Hospital, Zhengzhou, China

**Keywords:** esophageal squamous cell carcinoma, circPVT1, miR-30a-5p, FZD3, β-catenin, ferroptosis

## Abstract

**Background:**

CircPVT1 is demonstrated to promote cancer progression in esophageal squamous cell carcinoma (ESCC). However, the role and potential functional mechanisms of circPVT1 in regulating 5-fluorouracil (5-FU) chemosensitivity remain largely unknown.

**Methods:**

ESCC cells resistant to 5-FU were induced with continuous increasing concentrations of 5-FU step-wisely. A cell counting kit-8 assay was used to analyze the viability of ESCC cells. LDH release assay kit was used to evaluate the cytotoxicity. RT-qPCR was used to assess the expression level of non-coding RNAs and cDNAs. Luciferase was used to confirm the interaction between non-coding RNAs and targets. Western blotting was used to detect the expression of downstream signaling proteins. Flow cytometry and ferroptosis detection assay kit were utilized to measure the ferroptosis of ESCC cells.

**Results:**

CircPVT1 was significantly upregulated in ESCC cells resistant to 5-FU. Knockdown of circPVT1 enhanced the 5-FU chemosensitivity of ESCC cells resistant to 5-FU by increasing cytotoxicity and downregulating multidrug-resistant associated proteins, including P-gp and MRP1. Luciferase assay showed that circPVT1 acted as a sponge of miR-30a-5p, and Frizzled3 (FZD3) was a downstream target of miR-30a-5p. The enhanced 5-FU chemosensitivity by circPVT1 knockdown was reversed with miR-30a-5p inhibitor. Besides, the increased 5-FU chemosensitivity by miR-30a-5p mimics was reversed with FZD3 overexpression. Furthermore, knockdown of circPVT1 increased ferroptosis through downregulating p-β-catenin, GPX4, and SLC7A11 while miR-30a-5p inhibition and FZD3 overexpression reversed the phenotype by upregulating p-β-catenin, GPX4, and SLC7A11.

**Conclusions:**

These results suggested a key role for circPVT1 in ESCC 5-FU-chemosensitivity in regulating the Wnt/β-catenin pathway and ferroptosis *via* miR-30a-5p/FZD3 axis, which might be a potential target in ESCC therapy.

## Introduction

Esophageal carcinoma (EC) is a highly malignant cancer derived from esophageal epithelial cells, ranking as the tenth most prevalent cancer worldwide (1). EC includes two primary pathological types: esophageal adenocarcinoma (EAC) and esophageal squamous cell carcinoma (ESCC) (2). In Asian countries, ESCC is the leading cause of EC in Asian countries (3). Although advances in multidisciplinary treatment have improved the prognosis of ESCC patients, most patients will eventually yield to this malignancy, with a 5-year relative survival rate of < 20% (2). Therefore, it is urgent to search for effective novel targets to facilitate clinical treatment strategies for ESCC.

In recent years, compelling evidence has demonstrated the crucial roles of circular non-coding RNAs (circRNAs) in the pathogenesis and progression of ESCC (4). CircRNAs are reported to participate in various regulatory mechanisms, such as ceRNAs, protein interactions, and gene transcriptional and translational regulation in ESCC (4). Li et al. revealed that circ_0023984 exerted an oncogenic role in ESCC progression *via* miR-433-3p/REV3L axis (5). Wang et al. found that circ_0087378 inhibited the tumorigenesis and progression of ESCC through acting as a competing endogenous RNA and upregulating E2F3 expression (6). Wang et al. demonstrated that circ-ZDHHC5 promoted ESCC progression by sponging miR-217 with ZEB1 (7). In recent years, a novel circRNA, namely circPVT1, was reported to enhance the malignant phenotypes of ESCC, including proliferation and invasion, through regulating miR-4663/Pax and PPAR axis (8). However, it remains largely unknown whether circPVT1 plays a modulating role in the chemosensitivity of ESCC to 5-fluorouracil (5-FU).

The Frizzled (FZD) family is transmembrane receptors of the Wnt signaling pathway with 10 isoforms (FZD1-10) (9). Various studies have validated that the FZD family plays a crucial role in tumor development and progression (10–12). Several reports suggested that FZD3 was ungulated in cancers and promoted the proliferation and invasion of cancer cells *via* the Wnt/β-catenin pathway (13–15). However, a recent report suggested that FZD3 was downregulated in renal cell carcinoma and downregulation of FZD3 abolished the inhibitory effect of miR-340 knockdown on cell proliferation, migration, and invasion (16). This indicated a controversial role of FZD3 in malignancies. A previous study revealed that FZD3 was upregulated significantly in EAC, but the expression pattern and potential mechanisms of FZD3 in ESCC remain unveiled.

In this study, we verified a novel circPVT1 was significantly overexpressed in ESCC with chemoresistance 5-FU. In mechanisms, knockdown of circPVT1 enhanced 5-FU chemosensitivity of ESCC cells resistant to 5-FU *via* the miR-30a-5p/FZD3 axis. Our results demonstrated that circPVT1 plays an essential role in regulating ESCC chemosensitivity and may act as a potential target in the treatment of ESCC.

## Materials and Methods

### Ethics

The protocol of the present study was reviewed and approved by the Ethics Committee of Medical Research, Henan Provincial People’s Hospital. All the animal experiments were performed according to the guidelines of the Institution Animal Care and Use Committee.

### Cell Lines and Reagents

The normal human esophageal epithelial cells (HEEC) and ESCC cell lines, including EC9706 and KYSE70, were purchased from the Cell Bank of the Chinese Academy of Sciences (Chinese Academy of Sciences, Shanghai, China). Cells were maintained in DMEM (Invitrogen; Thermo Fisher Scientific, Inc.) containing 10% fetal bovine serum (Invitrogen; Thermo Fisher Scientific, Inc.), penicillin, and streptomycin (100 U/ml; Invitrogen; Thermo Fisher Scientific, Inc.) in a 5% CO2 humidified incubator (Thermo Fisher Scientific, Inc.) at 37°C. EC9706-5-Fu cell line was generated by incubating EC9706 cells with an increasing level of 5-FU (Sigma-Aldrich, St. Louis, MO, USA) and OX (Sigma-Aldrich) until the concentration reached 5 μM for OX and 5 μM for 5-FU. Resistant cell lines were maintained under constant treatment with the drug for daily cultivation until cells reached the growth phase.

### Reactive Oxygen Species Measurement

Intracellular ROS level was detected by staining cells with 2’, 7’-dichlorofluorescein diacetate (DCFH-DA) (GENMED, GMS10016.2) following the manufacturer’s instructions. The DCFH-DA signal was detected with a FACSCalibur flow cytometer, and the data were analyzed using FlowJo7.6.1 software.

### Cell Cytotoxicity Assay

Cell cytotoxicity was determined with a lactate dehydrogenase (LDH) release assay kit (Beyotime Biotechnology, Haimen, China), according to the manufacturers’ instructions. Briefly, two sets of controls were used, one as the spontaneous LDH release control (Min) and the other as the maximum LDH release control (Max) using lysis buffer. The supernatant was collected and mixed with the reaction mixture. After incubation for 30 min, the absorbance (A) was measured at 490 nm with a reference wavelength of 630 nm.

### Reverse Transcription-Quantitative PCR Analysis

Total RNA was isolated using TRIzol^®^ reagent (Invitrogen; Thermo Fisher Scientific, Inc.) according to the manufacturer’s instructions. Then, Nanodrop (Thermo Fisher Scientific, Inc.) was used to determine the concentration and purity of RNA. RNA was reverse-transcribed into cDNA with PrimeScript RT master mix (cat. no. RR036A; Takara Bio, Inc.) as the manufacturer’s protocol. LightCycler 96 system (Roche Diagnostics GmbH) was used to conduct qPCR with SYBR Premix Ex Taq II kit (cat. no. DRR081A; Takara Bio, Inc.). The qPCR conditions were as follows: 95°C for 10 minutes (min), 95°C for 10 seconds (sec), and 60°C for 60 sec, with a total of 40 cycles. The relative expression level of RNAs was calculated by referring to 2 ^-ΔΔCt^ methods. The primer sequence was detailed in **Table 1**.

**Table 1:** Specific RNAs primers for qRT-PCR analysis.

Gene	Primer	Sequence (5’-3’)
U6	Forward	CGCTTCGGCAGCACATATAC
	Reverse	AAATATGGAACGCTTCACGA
GAPDH	Forward	TCAAGAAGGTGGTGAAGCAGG
	Reverse	TCAAAGGTGGAGGAGTGGGT
hsa-miR-30a-5p	Forward	TGCGCTGTAAACATCCTCGACT
	Reverse	CCAGTGCAGGGTCCGAGGTATT
circPVT1	Forward	TGCCAGGACACTGAGATTTG
	Reverse	TTCCCCAGACCACTGAAGAT

### Cell Transfection

Small hairpin RNAs (shRNAs) targeting circPVT1 were designed and cloned into the GV102 vector (hU6-MCS-CMV-GFP-SV40-Neomycin) (GeneChem). EC cells (2 x 10^5^) were seeded in six-well plates and cultivated until reaching 70% confluency. Then, plasmids were transfected were transfected into EC cells using Lipofectamine™ 3000 (cat. no. L3000008; Invitrogen; Thermo Fisher Scientific, Inc.), according to the manufacturer’s protocols. The plates were incubated in a 5% CO_2_ incubator at 37°C for 24-72 h. Then, the cells were collected for subsequent experiments, and the transfection efficiency was verified by reverse transcription quantitative polymerase chain reaction (RT-qPCR).

### Cell Counting Kit-8 Assay

Cellular viability was determined by Cell Counting Kit-8 (Beyotime Biotechnology). Briefly, 100 μL cells per well were plated into 96-well plates. After the corresponding reagents treatment, 10 μL CCK8 solution was incubated with cell medium for 2 hours at 37°C. The absorbance of each well was detected at 450 nm by Multiskan FC Microplate spectrophotometer.

### Transwell Assays

Cells were seeded into Transwell migration chambers with a pore size of 8 µm (Corning, New York, NY, USA). The upper chamber was precoated with 50 µl of 1:8 diluted Matrigel (Corning) for the invasion assay. All these experiments were performed in triplicate.

### Wound Healing Assay

EC cells (5× 10^5^ cells/well) were seeded into 6-well plates (Corning) with 10 μg/mL mitomycin C (Biochempartner, Shanghai, China) for 3 hours. Wounds were made by scratching the adherent cells on the plate with a sterile 200 μL pipette tip (12 hours after seeding), replaced with fresh culture medium and then cultured for 24 hours. The migration ability was evaluated by analyzing the migration of the cells into the wounded area. All these experiments were performed in triplicate.

### Luciferase Reporter Assay

Circ-PVT1 vector or circPVT1-mutant (MUT) vector was constructed with or without a 3′-untranslated region binding site for miR-30a-5p using pmirGLO vector (Promega Corporation). FZD3-WT vector or FZD3-MUT vector was constructed similarly. Cells were co-transfected with miRNA mimics, or NC mimics, along with luciferase reporter vector as described above. After 48 h, luciferase assays were conducted to analyze the luciferase activity using the Dual-Luciferase Reporter Assay Kit (Promega, Madison, WI, USA) with a Dual-Luciferase Reporter Assay System (cat. no. E1910; Promega Corporation).

### Evaluation of Fe2+ Concentration and the Levels of MDA and GSH

An iron colorimetric assay kit (ScienCell, Cat: 8448) was applied to detect the intracellular ferrous iron levels according to the manufacturer’s instructions. A malondialdehyde (MDA) assay kit (Nanjing Jiancheng Bioengineering Institute, Nanjing, China) was utilized to determine the level of lipid peroxidation according to the manufacturer’s protocol. Total glutathione (GSH) was detected by the GSH cyclic reductase assay following the previous description (17).

### Protein Extraction and Western Blot Analysis

Cell proteins were extracted using RIPA buffer (cat. no. P0013B; Beyotime Institute of Biotechnology) containing EDTA-free protease inhibitor cocktail (cat. no. 04693159001; Roche Diagnostics GmbH). The concentration of proteins was measured with a BCA assay kit (Thermo Fisher Scientific). The proteins were separated with 8%-12% SDS-PAGE and then transferred to PVDF membranes. Then the membranes were blocked with 5% nonfat milk. Next, the membranes were incubated with primary antibodies at a dilution ratio of 1:1000 at 4°C overnight. Subsequently, the membranes were incubated with horseradish peroxidase-conjugated secondary antibodies at a dilution ratio of 1:5000 at room temperature for 1 h. Afterwards, the target proteins were visualized with enhanced chemiluminescence (cat. no. 407207; EMD Millipore; Merck KGaA) imaging system (Tanon Science and Technology Co., Ltd.).

### Immunofluorescence Staining

Cells were seeded onto Poly-lysine (cat: P4707, Millipore Sigma, Darmstadt, Germany) coated cover glasses. 1–2 days later, cells were washed with PBS three times at room temperature and then fixed with 4% paraformaldehyde Fix Solution (cat: E672002, Sangon, Shanghai, China) for 15 min. Next, the fixed cells were permeabilized with 0.2% Triton X-100 (cat: E-IR-R122, Elabscience Biotechnology, Wuhan, China) for 10 min at room temperature. Sections were incubated with γH2AX (cat: ab26350, Abcam Cambridge Science Park, Cambridge, UK) primary antibodies at 4°C overnight. The next day, sections were washed using PBS for 3 × 5 min at room temperature, and then incubated with 4’,6-diamidino-2-phenylindole (DAPI) dye (cat: D1306, ThermoFisher Scientific, Waltham, MA, USA) and fluorophore-conjugated secondary antibodies at room temperature for 2 h. Images were captured by the Leica microscope.

### ESCC Xenograft Model

BALB/c nude male mice of 4 weeks old were purchased from the Model Animal Research Center of Nanjing University (Nanjing, China). After one week of adaptive feeding, EC9706cells (3x10^6^) stably expressing sh-NC and sh-circPVT1, sh-NC+5-FU and sh-circPVT1+5-FU were subcutaneously were injected into the right flank of the nude mice in a serum-free DMEM medium. The tumor volumes were measured every week in the mice using the following formula: V = (length x width^2^)/2. After 4 weeks, tumor weight was measured.

### Statistical Analysis

All data generated were performed in triplicate and were analyzed using the GraphPad Prism v8.0 (GraphPad Software, Inc.). Student’s t-test, χ2 test, or one-way ANOVA analysis was performed appropriately. A significant difference was considered at P<0.05. Data are presented as mean ± SEM from three independent experiments.

## Results

### Expression of circPVT1 in ESCC Cells and ESCC Cells With Chemoresistance to 5-FU

**Figure 1:**
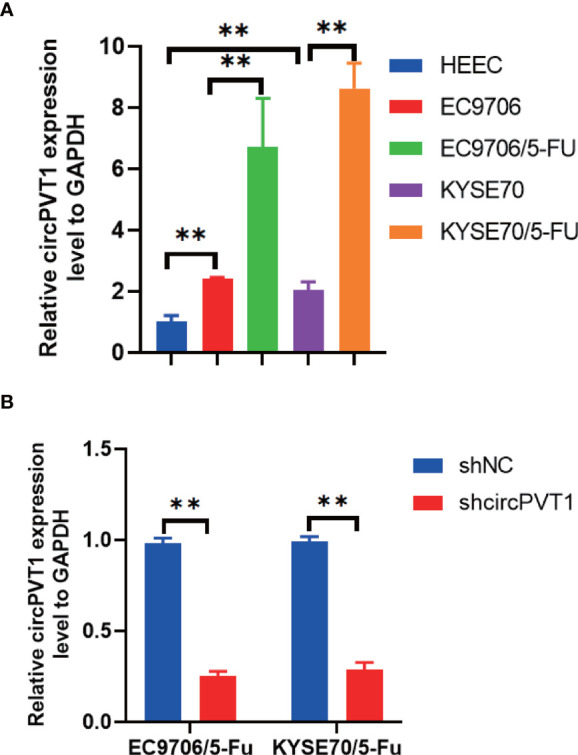
Expression of circPVT1 in ESCC cells and ESCC cells with chemoresistance to 5-FU. **(A)** The expression level of circPVT1 was measured in HEEC, EC9706, KYSE70, and EC9706/5-Fu and KYSE70/5-Fu cells using RT-qPCR. **(B)** RT-qPCR to detect circPVT1 expression in EC9706/5-Fu and KYSE70/5-Fu cells. Data are presented as mean ± SEM; n≥3. **P < 0.01.

We firstly measured the expression of circPVT1 in ESCC cells and ESCC cells with chemoresistance to 5-FU (EC9706/5-Fu and KYSE70/5-Fu). The results showed that circPVT1 was significantly upregulated in ESCC cells with chemoresistance to 5-FU compared with ESCC cells (**Figure 1A**). This indicated circPVT1 may play a key role in maintaining the chemoresistance to 5-FU of ESCC cells.

### Downregulation of CircPVT1 Promoted Chemosensitivity of ESCC Cells

**Figure 2:**
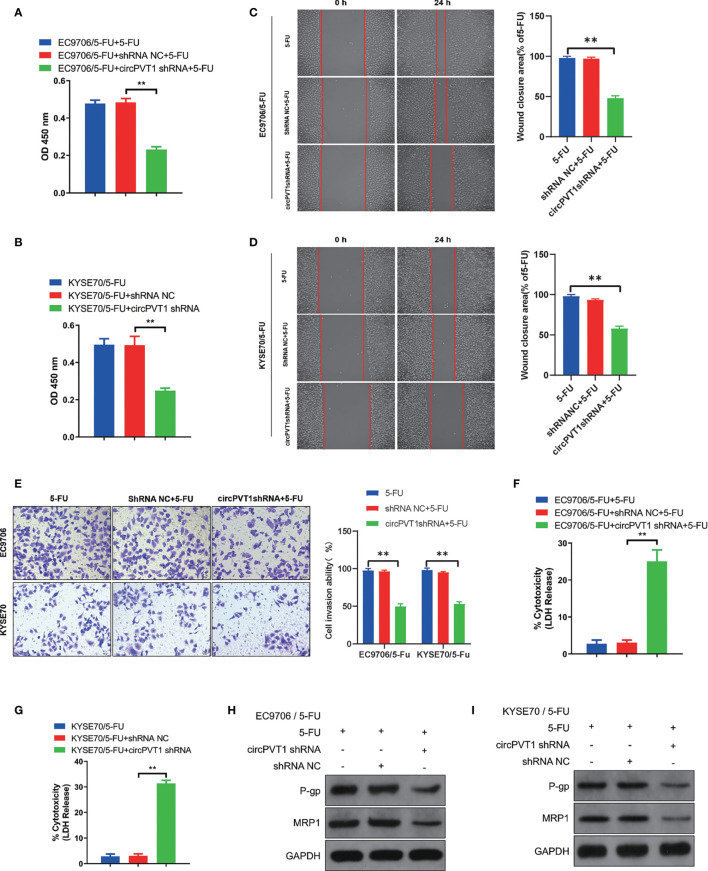
Downregulation of circPVT1 promoted chemosensitivity of ESCC cells. **(A)**Cell viability was quantified using Cell Counting Kit-8 assays after transfecting circPVT1 shRNA in EC9706/5-Fu cells. **(B)** Cell viability was quantified using Cell Counting Kit-8 assays after transfecting circPVT1 shRNA into KYSE70/5-Fu cells. **(C, D)** Wound healing assay after transfecting circPVT1 shRNA into EC9706/5-Fu and KYSE70/5-Fu cells. **(E)** Transwell assays revealed that the invasion capacity after transfecting circPVT1 shRNA into EC9706/5-Fu and KYSE70/5-Fu cells. **(F)** Cell cytotoxicity was quantified using LDH release assays after transfecting circPVT1 shRNA in EC9706/5-Fu cells. **(G)** Cell cytotoxicity was quantified using LDH release assays after transfecting circPVT1 shRNA into KYSE70/5-Fu cells. **(H)** The expression levels of P-gp and MRP1 in EC9706/5-Fu cells were measured with western blotting. **(I)** The expression levels of P-gp and MRP1 in KYSE70/5-Fu cells were measured with western blotting. Data are presented as mean ± SEM; n≥3. **P < 0.01.

We designed one shRNA specifically targeting circPVT1 and transfected it into EC9706/5-Fu and KYSE70/5-Fu cells. The circPVT1 expression was significantly reduced after transfection (**Figure 1B**). Then, we measured the cell viability after transfection. Our results showed that cell viability was inhibited after the knockdown of circPVT1 in EC9706/5-Fu and KYSE70/5-Fu cells (**Figures 2A, B**). Furthermore, we performed the wound healing assays on the cells and found that the migration ability was significantly decreased upon circPVT1 knockdown (P < 0.05) (**Figures 2C, D**). Next, transwell assays were performed to determine effect of circPVT1 on the invasion. As shown in **Figure 2E**, the invasive cells were significantly reduced with the knockdown of circPVT1. In addition, the results showed that the knockdown of circPVT1 increased cell cytotoxicity of EC9706/5-Fu and KYSE70/5-Fu cells (**Figures 2F, G**). We further detected the associated proteins of chemoresistance. The results showed that knockdown of circPVT1 significantly decreased the expression of P-gp and MRP1 in EC9706/5-Fu and KYSE70/5-Fu cells (**Figures 2H, I**). Together, these results indicated that downregulation of circPVT1 promoted chemosensitivity of ESCC cells.

### CircPVT1 Acted as a Sponge of MiR-30a-5p to Regulate the Chemosensitivity of ESCC Cells

**Figure 3:**
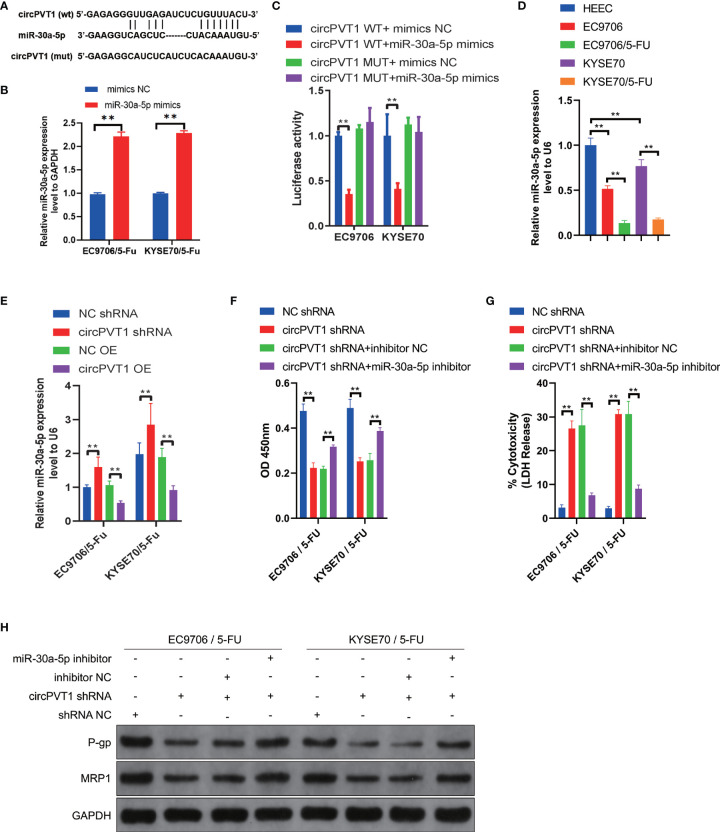
circPVT1 acted as a sponge of miR-30a-5p to regulate the chemosensitivity of ESCC cells. **(A)** The potential binding sites of miR-30a-5p and circPVT1. **(B)** The expression of miR-30a-5p in EC9706/5-Fu and KYSE70/5-Fu cells after transfection with miR-30a-5p mimics. **(C)** miR-30a-5p was validated as a direct target of circPVT1 using dual-luciferase reporter assays in EC9706 and KYSE70 cells. **(D)** The expression level of miR-30a-5p was detected in HEEC, EC9706, KYSE70, and EC9706/5-Fu and KYSE70/5-Fu cells using RT-qPCR. **(E)** The expression level of miR-30a-5p was measured after transfected with circPVT1 shRNA and circPVT1 overexpression plasmid in EC9706/5-Fu and KYSE70/5-Fu cells. **(F)** Cell viability was quantified using Cell Counting Kit-8 assays after transfecting circPVT1 shRNA or the combination of circPVT1 shRNA and miR-30a-5p inhibitor into EC9706/5-Fu and KYSE70/5-Fu cells. **(G)** Cell cytotoxicity was quantified using LDH release assays after transfecting circPVT1 shRNA or the combination of circPVT1 shRNA and miR-30a-5p inhibitor into EC9706/5-Fu and KYSE70/5-Fu cells. **(H)** The expression levels of P-gp and MRP1 were measured after transfecting circPVT1 shRNA or the combination of circPVT1 shRNA and miR-30a-5p inhibitor into EC9706/5-Fu and KYSE70/5-Fu cells *via* western blotting. Data are presented as mean ± SEM; n≥3. **P < 0.01.

Emerging studies have shown that circRNAs are involved in regulating tumor progression mainly *via* miRNA sponging (18). To investigate the functional mechanisms of circPVT1, we performed bioinformatics analyses to predict the potential targets. Combined with the predictive results of Starbase (https://starbase.sysu.edu.cn), miR-30a-5p was suggested as the possible complementary miRNA binding to circPVT1 (**Figure 3A**). To determine the interaction between circPVT1 and miR-30a-5p, dual−luciferase reporter gene assays were performed. The expression of miR-30a-5p in EC9706/5-Fu and KYSE70/5-Fu cells was up-regulated by transfection with miR-30a-5p mimics (**Figure 3B**). The luciferase assays showed that cells co-transfected with circPVT1 WT and miR-30a-5p mimics exhibited reduced activity compared with that in the control groups (**Figure 3C**). Next, the expression level of miR-30a-5p was determined in ESCC cells and ESCC cells with chemoresistance to 5-FU. The results showed that miR-30a-5p was decreased in ESCC cells compared with normal esophageal epithelial cells (**Figure 3D**). The expression level of miR-30a-5p was much lower in EC9706/5-Fu and KYSE70/5-Fu cells than in EC9706 and KYSE70 cells (**Figure 3D**). Then, we detected the expression level of miR-30a-5p after transfection with circPVT1 shRNA and overexpression plasmid in EC9706/5-Fu and KYSE70/5-Fu cells. The results revealed that downregulation of circPVT1 significantly increased the expression of miR-30a-5p, and upregulation of circPVT1 decreased miR-30a-5p remarkably (**Figure 3E**). The cell viability and cytotoxicity were subsequently examined after co-transfection with circPVT1 shRNA and miR-30a-5p inhibitor. The data showed that suppressed cell viability and increased cell cytotoxicity with circPVT1 knockdown were reversed after co-transfection with circPVT1 shRNA and miR-30a-5p inhibitor (**Figures 3F, G**). Furthermore, we detected the associated proteins of chemoresistance after co-transfection with circPVT1 shRNA and miR-30a-5p inhibitor. The results showed that a decrease of the expression of P-gp and MRP1 induced by circPVT1 shRNA was recovered after co-transfection with circPVT1 shRNA and miR-30a-5p inhibitor (**Figure 3H**). Collectively, these results manifested that circPVT1 regulated chemosensitivity of ESCC cells *via* sponging miR-30a-5p.

### MiR-30a-5p Increased Chemosensitivity of ESCC Cells *via* Downregulating FZD3

**Figure 4:**
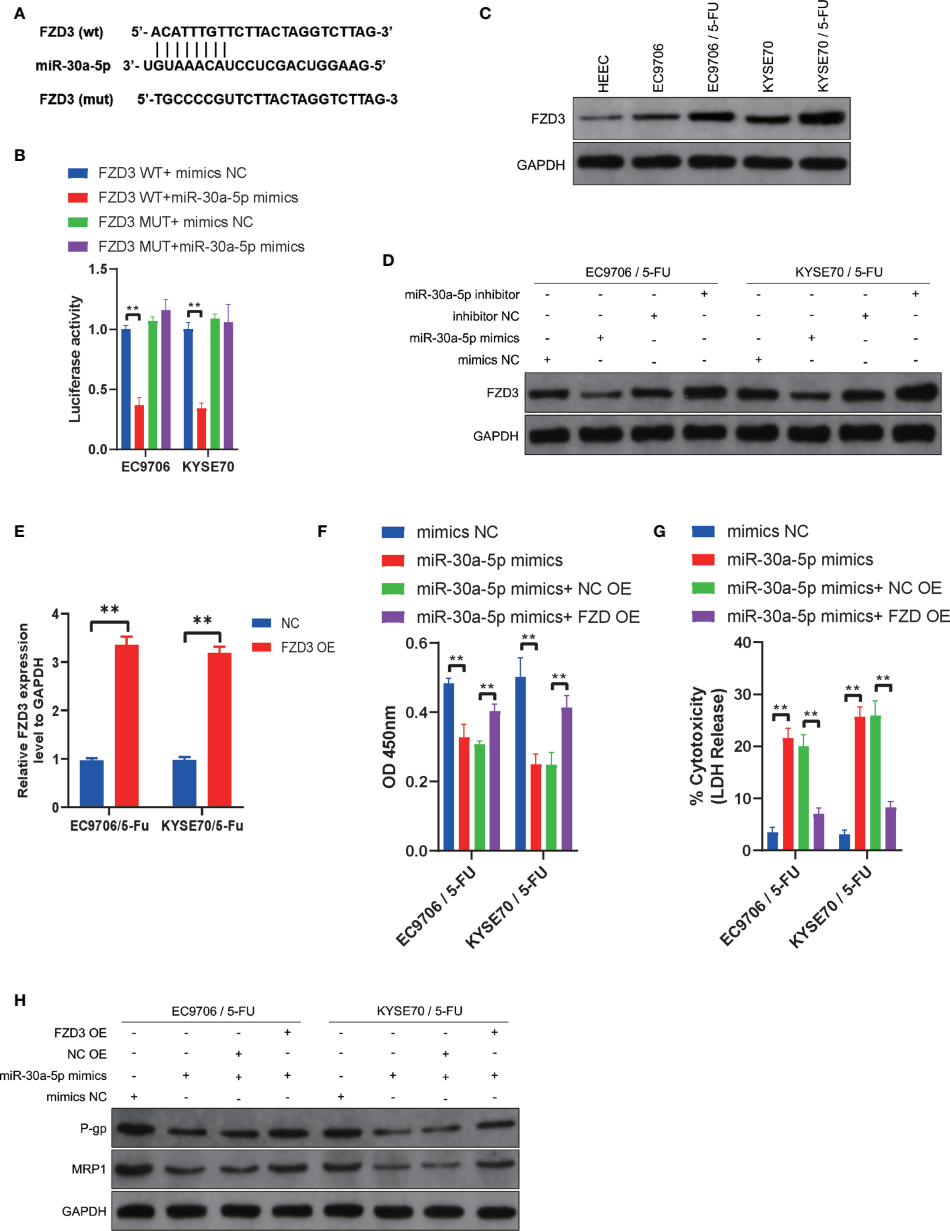
miR-30a-5p increased chemosensitivity of ESCC cells *via* downregulating FZD3. **(A)** The potential binding sites of miR-30a-5p and FZD3. **(B)** FZD3 was validated as a direct target of miR-30a-5p using dual-luciferase reporter assays in EC9706 and KYSE70 cells. **(C)** The expression level of FZD3 was measured in HEEC, EC9706, KYSE70, EC9706/5-Fu, and KYSE70/5-Fu cells *via* western blotting. **(D)** The expression level of FZD3 was measured after transfecting miR-30a-5p mimics or miR-30a-5p inhibitor into EC9706/5-Fu and KYSE70/5-Fu cells *via* western blotting. **(E)** The expression of FZD3 in EC9706/5-Fu and KYSE70/5-Fu cells after transfecting overexpression plasmid. **(F)** Cell viability was quantified using Cell Counting Kit-8 assays after transfecting miR-30a-5p mimics or combining miR-30a-5p mimics and FZD3 overexpression plasmid into EC9706/5-Fu and KYSE70/5-Fu cells. **(G)** Cell cytotoxicity was quantified using LDH release assays after transfecting miR-30a-5p mimics or combining miR-30a-5p mimics and FZD3 overexpression plasmid into EC9706/5-Fu and KYSE70/5-Fu cells. **(H)** The expression levels of P-gp and MRP1 were measured after transfecting miR-30a-5p mimics or the combination of miR-30a-5p mimics and FZD3 overexpression plasmid into EC9706/5-Fu and KYSE70/5-Fu cells. Data are presented as mean ± SEM; n≥3. **P < 0.01.

We further investigated the potential downstream targets of miR-30a-5p in the regulation chemosensitivity of ESCC cells. Bioinformatics analyses were used to predict the potential targets of miR-30a-5p with miRSystem (http://mirsystem.cgm.ntu.edu.tw/index.php). FZD3 was analyzed to be a target of miR-30a-5p (**Figure 4A**). Luciferase assays showed that cells co-transfected with FZD3 WT and miR-30a-5p mimics exhibited reduced activity compared with that in the control groups (**Figure 4B**). Next, the expression level of FZD3 was measured in ESCC cells and ESCC cells with chemoresistance to 5-FU. The results showed that FZD3 was increased in ESCC cells compared with normal esophageal epithelial cells (**Figure 4C**). The expression level of FZD3 was much higher in EC9706/5-Fu and KYSE70/5-Fu cells compared with EC9706 and KYSE70 cells (**Figure 4C**). Then, we detected the expression level of FZD3 after transfection with miR-30a-5p inhibitor and miR-30a-5p mimics. The results revealed that downregulation of miR-30a-5p significantly increased the expression of FZD3, and upregulation of miR-30a-5p decreased FZD3 remarkably (**Figure 4D**). After transfecting overexpression plasmid, FZD3 was upregulated in EC9706/5-Fu and KYSE70/5-Fu cells (**Figure 4E**). Subsequently, the cell viability and cytotoxicity were examined after co-transfection with miR-30a-5p mimics and FZD3 overexpression plasmid. The data showed that suppressed cell viability and increased cell cytotoxicity with miR-30a-5p mimics were reversed after co-transfection with miR-30a-5p mimics and FZD3 overexpression plasmid (**Figures 4F, G**). Subsequently, we detected the associated proteins of chemoresistance after co-transfection with miR-30a-5p mimics and FZD3 overexpression plasmid. The results showed that a decrease of the expression of P-gp and MRP1 induced by miR-30a-5p mimics was recovered after co-transfection with miR-30a-5p mimics and FZD3 overexpression plasmid (**Figure 4H**). Collectively, these results revealed that miR-30a-5p modulated the chemosensitivity of ESCC cells *via* targeting FZD3.

### CircPVT1 Regulated Chemosensitivity of ESCC Cells Through Ferroptosis and Wnt/β-Catenin Pathways *via* MiR-30a-5p/FZD3

**Figure 5:**
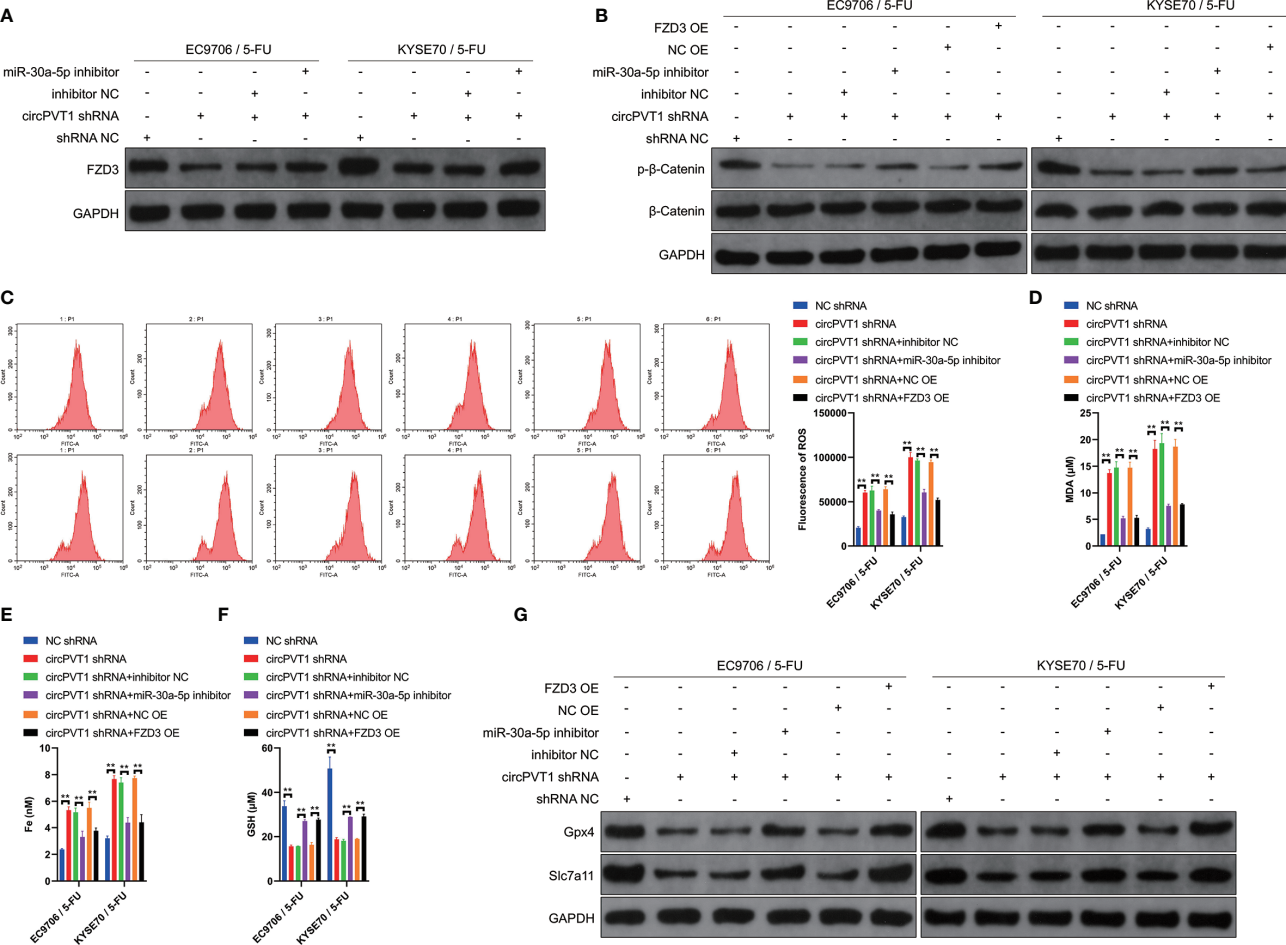
circPVT1 regulated chemosensitivity of ESCC cells through ferroptosis and Wnt/β-catenin pathways *via* miR-30a-5p/FZD3. **(A)** The expression level of FZD3 was measured after transfecting circPVT1 shRNA or the combination of circPVT1 shRNA and miR-30a-5p inhibitor into EC9706/5-Fu and KYSE70/5-Fu cells *via* western blotting. **(B)** The expression level of β-catenin and p-β-catenin was measured after transfecting circPVT1 shRNA or the combination of circPVT1 shRNA and miR-30a-5p inhibitor or the combination of circPVT1 shRNA and FZD3 overexpression plasmid into EC9706/5-Fu and KYSE70/5-Fu cells *via* western blotting. **(C)** ROS level was detected (left) and quantified (right) after transfecting circPVT1 shRNA or the combination of circPVT1 shRNA and miR-30a-5p inhibitor or the combination of circPVT1 shRNA and FZD3 overexpression plasmid into EC9706/5-Fu and KYSE70/5-Fu cells *via* flow cytometer. **(D)** The level of MDA was measured after transfecting circPVT1 shRNA or the combination of circPVT1 shRNA and miR-30a-5p inhibitor or the combination of circPVT1 shRNA and FZD3 overexpression plasmid into EC9706/5-Fu and KYSE70/5-Fu cells. **(E)** The level of iron was measured after transfecting circPVT1 shRNA or the combination of circPVT1 shRNA and miR-30a-5p inhibitor or the combination of circPVT1 shRNA and FZD3 overexpression plasmid into EC9706/5-Fu and KYSE70/5-Fu cells. **(F)** The level of GSH was measured after transfecting circPVT1 shRNA or the combination of circPVT1 shRNA and miR-30a-5p inhibitor or the combination of circPVT1 shRNA and FZD3 overexpression plasmid into EC9706/5-Fu and KYSE70/5-Fu cells. **(G)** The expression levels of GPX4 and SLC7A11 were measured after transfecting circPVT1 shRNA or the combination of circPVT1 shRNA and miR-30a-5p inhibitor or the combination of circPVT1 shRNA and FZD3 overexpression plasmid into EC9706/5-Fu and KYSE70/5-Fu cells *via* western blotting. Data are presented as mean ± SEM; n≥3. **P < 0.01.

We then investigated the potential mechanisms of circPVT1 in regulating the chemosensitivity of ESCC cells *via* the miR-30a-5p/FZD3 axis. We first examined the expression levels of FZD3 after transfection with miR-30a-5p inhibitor and circPVT1 shRNA, respectively. The results showed that circPVT1 knockdown significantly decreased the expression of FZD3, while co-transfection of circPVT1 shRNA and miR-30a-5p inhibitor partially recovered the expression of FZD3 (**Figure 5A**). This validated that circPVT1 regulated the chemosensitivity of ESCC cells *via* miR-30a-5p/FZD3 axis. We further detected the effect of circPVT1/miR-30a-5p/FZD3 axis on the Wnt/β-catenin pathway. The results showed that circPVT1 knockdown significantly inhibited the Wnt/β-catenin pathway in EC9706/5-Fu and KYSE70/5-Fu cells (**Figure 5B**). Notably, miR-30a-5p inhibitor and FZD3 overexpression plasmid remarkably reversed the inhibited Wnt/β-catenin pathway (**Figure 5B**). Previous studies suggested that ferroptosis played a fundamental role in maintaining the status of chemoresistance. We examined ROS levels in the downstream of circPVT1/miR-30a-5p/FZD3 axis in EC9706/5-Fu and KYSE70/5-Fu cells. The results revealed that circPVT1 knockdown statistically increased ROS level of EC9706/5-Fu and KYSE70/5-Fu cells, which was partially recovered after co-transfection miR-30a-5p inhibitor and FZD3 overexpression plasmid (**Figure 5C**). We also determined the expression levels of ferroptosis-associated parameters. The results showed that expression levels of MDA and Fe were significantly upregulated with circPVT1 knockdown, which was then partially decreased after co-transfection with circPVT1 shRNA and miR-30a-5p inhibitor or co-transfection with circPVT1 shRNA and FZD3 overexpression plasmid (**Figures 5D, E**). On the contrary, the expression levels of GSH were significantly downregulated with circPVT1 knockdown, which was then partially increased after co-transfection with circPVT1 shRNA and miR-30a-5p inhibitor or co-transfection with circPVT1 shRNA and FZD3 overexpression plasmid (**Figure 5F**). Furthermore, we detected Glutathione Peroxidase 4 (GPX4) and Solute Carrier Family 7 Member 11 (SLC7A11) in EC9706/5-Fu and KYSE70/5-Fu cells. The results showed that circPVT1 knockdown significantly decreased the expression levels of GPX4 and SLC7A11, which were then partially increased after co-transfection with circPVT1 shRNA and miR-30a-5p inhibitor or co-transfection with circPVT1 shRNA and FZD3 overexpression plasmid (**Figure 5G**). Collectively, circPVT1 regulated chemosensitivity of ESCC cells through ferroptosis and Wnt/β-catenin pathways *via* miR-30a-5p/FZD3.

### Downregulation of CircPVT1 Enhanced Chemosensitivity *In Vivo*

**Figure 6:**
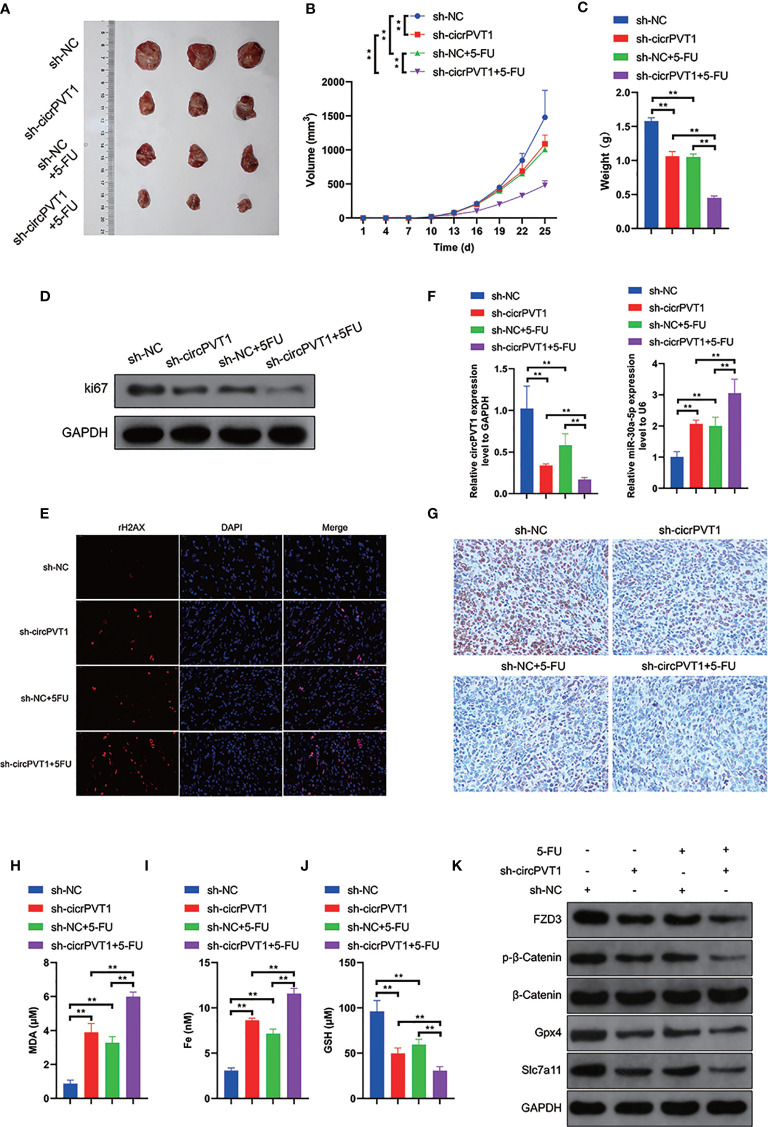
Downregulation of circPVT1 enhanced chemosensitivity *in vivo*. **(A)** EC9706 cells transfected with circPVT1 shRNAs, 5-FU, and the combination of circPVT1 shRNAs and 5-FU were injected subcutaneously into the flanks of nude mice. Tumors were extracted after 4 weeks. **(A)** An image is illustrating xenograft tumors harvested from various treatment groups. **(B)** Tumor volumes of mice were measured every 3 days. **(C)** The tumor weights of mice were compared among the groups after 4 weeks. **(D)** The protein expression level of ki67 among the four groups. **(E)** Immunofluorescence showed the level of γH2AX (red dots) among the four groups. **(F)** The expression of circPVT1 and miR-30a-5p was detected among the groups with RT-qPCR. **(G)** The expression of FZD3 was detected among the groups with immunohistochemistry. **(H)** The level of MDA was measured among the groups. **(I)** The level of iron was measured among the groups. **(J)** The level of GSH was measured among the groups. **(K)** The expression levels of FZD3, β-catenin, p-β-catenin, GPX4, and SLC7A11 were detected among the groups *via* western blotting. Data are presented as mean ± SEM; n≥3. **P < 0.01.

Finally, we evaluated the effect of circPVT1 knockdown *in vivo*. The results showed that circPVT1 knockdown remarkably inhibited tumor growth, compared with the treatment with 5-FU (**Figures 6A** **–C**). Notably, the tumor growth was much slower with the combination of circPVT1 shRNA and 5-FU than that of treatment with 5-FU alone (**Figures 6A** **–C**). Also, we found that the combination of circPVT1 shRNA and 5-FU group showed the lowest expression of ki67 (**Figure 6D**). In addition, the level of γH2AX, a marker of DNA damage, was significantly increased in combination of circPVT1 shRNA and 5-FU group (**Figure 6E**). These indicated knockdown of circPVT1 and 5-FU could inhibit the tumor growth. We further investigated the expression of circPVT1 shRNA and miR-30a-5p in tumors extracted from mice. The results showed that circPVT1 was significantly reduced in mice with circPVT1 knockdown, but miR-30a-5p was remarkably increased with downregulation of circPVT1 (**Figure 6F**). The results of pathology indicated that FZD3 was significantly decreased with circPVT1 knockdown (**Figure 6G**). We further detected the expression levels of ferroptosis-associated parameters. The results showed that expression levels of MDA and Fe were significantly upregulated with circPVT1 knockdown (**Figures 6H, I**), and that expression levels of GSH were downregulated considerably with circPVT1 knockdown (**Figure 6J**). Moreover, the protein levels of FZD3, β-catenin, GPX4, and SLC7A11 were all remarkably reduced in the circPVT1 knockdown group, especially in circPVT1 and 5-FU group (**Figure 6K**). Together, these results confirmed the downregulation of circPVT1 enhanced chemosensitivity *in vivo* through ferroptosis and Wnt/β-catenin pathways *via* miR-30a-5p/FZD3.

## Discussion

In this study, we found that circPVT1 was upregulated in ESCC cells with chemoresistance. In addition, downregulation of circPVT1 promoted chemosensitivity of ESCC cells *in vitro* and *in vivo*. Furthermore, circPVT1 positively modulated the expression of FZD3 *via* sponging miR-30a-5p. In sum, our study revealed that circPVT1 regulated the chemosensitivity of ESCC cells through ROS and Wnt/β-catenin pathways *via* miR-30a-5p/FZD3. This study is the first study to provide evidence that circPVT1 might be used as a chemosensitizing agent in ESCC.

Chemoresistance is emerging as a significant obstacle to the effective treatment of ESCC (19). Previous reports have validated that the chemoresistance rate of ESCC to 5‐FU is nearly 85% (20, 21). Various studies indicated that circRNAs played a crucial role in modulating the chemosensitivity of cancer cells to 5‐FU treatment (22, 23). Gao et al. found that circPARD3 promoted laryngeal squamous cell carcinoma progression and chemoresistance through the PRKCI-Akt-mTOR pathway (24). Hong et al. revealed that circCRIM1 functioned as a ceRNA sponge to promote nasopharyngeal carcinoma metastasis and docetaxel chemoresistance through upregulating FOXQ1 (25). Jian et al. found that circ_001680 affected the proliferation and migration of colorectal cancer and mediated its chemoresistance by regulating BMI1 through miR-340 (26). In addition, some other reports also found circRNAs modulated chemoresistance in pancreatic cancer, lung cancer, and osteosarcoma (27–29). However, only a few studies investigated the role of circRNAs in regulating chemoresistance in ESCC. CircPVT1 is derived from a long noncoding RNA region of oncogene PVT1, which is a cancer susceptibility locus (30). Several reports have demonstrated the vital role of circPVT1 in maintaining chemoresistance in cancers. For example, Zhu et al. found that overexpressed circPVT1 contributed to doxorubicin and cisplatin resistance of osteosarcoma cells by regulating ABCB1 (31). Wang et al. demonstrated that knockdown of circPVT1 elevated gastric cancer cisplatin sensitivity *via* sponging miR-152-3p (32). However, the role of circPVT1 in regulating chemoresistance in ESCC remains largely unknown. In this study, we found that circPVT1 played a fundamental role in regulating chemoresistance in ESCC. Knockdown of circPVT1 increased the chemosensitivity of ESCC cells to 5-FU. This emphasized the importance of circPVT1 in maintaining chemoresistance in ESCC.

It has been reported that FZD3 was upregulated in some cancers and promoted the proliferation and invasion of cancer cells *via* the Wnt/β-catenin pathway (13–15). However, a recent report suggested that FZD3 was downregulated in renal cell carcinoma and downregulation of FZD3 abolished the inhibitory effect of miR-340 knockdown on cell proliferation, migration, and invasion (16). This indicated a controversial role of FZD3 in malignancies. Here, we found that FZD3 was upregulated in ESCC and played a crucial role in maintaining the chemoresistance of ESCC to 5-FU. We also found that circPVT1 could regulate FZD3 through miRNA sponging. This indicated that FZD3 might be a potential therapeutic target in promoting chemosensitivity in ESCC. FZD3 is one of the transmembrane receptors of the Wnt signaling pathway. In accordance with these reports, we also found that downregulated FZD3 induced circPVT1 knockdown suppressed the activation of the Wnt/β-catenin pathway by reducing the expression of p-β-catenin.

Emerging studies have indicated that ferroptosis is closely associated the chemoresistance in malignancies. Zhang et al. found that CAF secreted miR-522 suppressed ferroptosis and promoted acquired chemoresistance in gastric cancer (33). Chan et al. revealed that MAP30 protein showed synergic activity with cisplatin against ovarian cancer *in vivo* by altering metabolism and inducing ferroptosis (34). Deng et al. found that miR-324-3p reversed cisplatin resistance by inducing GPX4-mediated ferroptosis in lung adenocarcinoma cell line A549 (35). However, the role of ferroptosis in regulating chemoresistance in ESCC remains largely obscure. Our study found that knockdown of circPVT1 promoted chemosensitivity in ESCC by increasing ferroptosis *via* downregulating GPX4 and SLC7A11. This may promote our understanding of ferroptosis in regulating chemoresistance.

## Conclusion

In conclusion, we found that circPVT1 was upregulated in ESCC cells with chemoresistance. In addition, our study revealed that circPVT1 regulated the chemosensitivity of ESCC cells through ROS and Wnt/β-catenin pathways *via* miR-30a-5p/FZD3. This provides evidence that circPVT1 might be a chemosensitizing agent in ESCC.

## Data Availability Statement

The original contributions presented in the study are included in the article/supplementary material. Further inquiries can be directed to the corresponding author.

## Ethics Statement

The protocol of the present study was reviewed and approved by the Ethics Committee of Medical Research, Henan Provincial People’s Hospital.

## Author contributions

LW and WY conceived this study. WY, JW, FM, ZZ, XJ, LX, and QZ performed *in vivo* and *in vitro* experiments and analyzed the data. WY wrote the manuscript. LW reviewed and modified the manuscript. All the authors read and approved the final manuscript.

## Funding

This study was supported by Key Science and Technology Projects in Henan Province (212102310669, 202102310457), Henan Province medical science and technology research plan joint construction project (LHGJ20200034) & “23456 Talent Project” of Henan Provincial People’s Hospital.

## Conflict of Interest

The authors declare that the research was conducted in the absence of any commercial or financial relationships that could be construed as a potential conflict of interest.

## Publisher’s Note

All claims expressed in this article are solely those of the authors and do not necessarily represent those of their affiliated organizations, or those of the publisher, the editors and the reviewers. Any product that may be evaluated in this article, or claim that may be made by its manufacturer, is not guaranteed or endorsed by the publisher.
